# Punching above their weight: a network to understand broader determinants of increasing life expectancy

**DOI:** 10.1186/s12939-018-0832-y

**Published:** 2018-08-13

**Authors:** Fran Baum, Jennie Popay, Toni Delany-Crowe, Toby Freeman, Connie Musolino, Carlos Alvarez-Dardet, Vinya Ariyaratne, Kedar Baral, Paulin Basinga, Mary Bassett, David M. Bishai, Mickey Chopra, Sharon Friel, Elsa Giugliani, Hideki Hashimoto, James Macinko, Martin McKee, Huong Thanh Nguyen, Nikki Schaay, Orielle Solar, Sundararaman Thiagarajan, David Sanders

**Affiliations:** 1 Southgate Institute for Health, Society & Equity, Flinders University, Adelaide, South Australia Australia; 2 Institute for Health Research, Lancaster University, Bailrigg, Lancaster UK; 3 The Observatory of Public Policies and Health, Center for Research in Epidemiology and Public Health, University of Alicante, Alicante, Spain; 4 Sarvodaya Shramadana Movement, Sarvodaya Headquarters “Damsak Mandira”, Moratuwa, Sri Lanka; 5 Department of Community Health Sciences, Patan Academy of Health Sciences, Kathmandu, Nepal; 6 Integrated Delivery Country Primary Health Care, Bill and Melinda Gates Foundation, Abuja, Nigeria; 7 New York City Department of Health and Mental Hygiene, Office of General Counsel, Long Island City, New York USA; 8 Population, Family, and Reproductive Health, Johns Hopkins University Bloomberg School of Public Health, Baltimore, Maryland USA; 9 World Bank, Washington, USA; 10 School of Regulation and Global Governance (RegNet), Australian National University, Canberra, Australian Capital Territory Australia; 11 College of Asia and the Pacific, Australian National University, Canberra, Australian Capital Territory Australia; 12 School of Medicine, Department of Pediatrics and Child Care, Federal University of Rio Grande do Sul, Porto Alegre, Brazil; 13 Health Economics and Epidemiology Research, School of Public Health, University of Tokyo, Tokyo, Japan; 14 Departments of Health Policy and Management and Community Health Sciences, UCLA Fielding School of Public Health, Los Angeles, California USA; 15 European Public Health, London School of Hygiene and Tropical Medicine, London, UK; 16 Department of Health Management and Organization, Hanoi Medical University, Hanoi, Vietnam; 17 Faculty of Community and Health, School of Public Health, University of the Western Cape, Bellville, Republic of South Africa; 18 Faculty of Medicine, Programa de Salud Ocupacional, Académico, Escuela de Salud Pública, Universidad de Chile, Santiago, Chile; 19 School of Health Systems Research, Tata Institute of Social Sciences, Mumbai, India; 20 School of Public Health, University of the Western Cape, Bellville, South Africa

**Keywords:** Life expectancy, Health equity, Social determinants of health, Politics of health, Gender equity, Civil society, Health improvement

## Abstract

**Background:**

Life expectancy initially improves rapidly with economic development but then tails off. Yet, at any level of economic development, some countries do better, and some worse, than expected – they either punch above or below their weight. Why this is the case has been previously researched but no full explanation of the complexity of this phenomenon is available.

**New research network:**

In order to advance understanding, the newly formed Punching Above Their Weight Research Network has developed a model to frame future research. It provides for consideration of the following influences within a country: political and institutional context and history; economic and social policies; scope for democratic participation; extent of health promoting policies affecting socio-economic inequities; gender roles and power dynamics; the extent of civil society activity and disease burdens.

**Conclusion:**

Further research using this framework has considerable potential to advance effective policies to advance health and equity.

## Background

In September 2017 the *Punching Above Their Weight Research Network* [1] was formed to advance thinking and research about why some countries do much better or much worse in terms of life expectancy than would be predicted by their economic status. It builds on previous research that has focused primarily on health sector performance. Previous attempts have not drawn adequately on an interdisciplinary approach and have thus failed to produce a sufficiently nuanced and holistic picture of the political, social, environmental and economic processes that drive good or poor performance in promoting population health and health equity.

The Preston curve has demonstrated that, in general, life expectancy initially improves rapidly with economic development but the improvement then tails off [2]. Yet, at any level of economic development, some countries do better, and some worse, than expected. What can we learn from those countries, or regions within countries, at all levels of development that deviate from the mean to punch either above or below their weight? (Table 1).

**Table 1:** Top five punching above their weight countries and bottom five at each level of development (2015)

Low income	Middle Income	High Income
Top performers (top row punches most above weight)		
Nepal	Honduras	Japan
Madagascar	Viet Nam	Spain
Rwanda	Nicaragua	Chile
Liberia	Bangladesh	Greece
Ethiopia	Solomon Islands	South Korea
Bottom performers (bottom row punches the most below weight)		
Central African Republic	Swaziland	Brunei Darussalam
Mali	Cote d’Ivoire	Saudi Arabia
South Sudan	Nigeria	Trinidad and Tobago
Chad	Angola	Qatar
Sierra Leone	Equatorial Guinea	Kuwait

Note: Performance is defined as the distance from the regression line linking life expectancy at birth (2015) [28] and gross domestic product (2015) [29]

It may be shocking news to many citizens of the United States that Costa Rica, with a Gross Domestic Product per capita a tenth of the US, outstrips them in life expectancy by one year [3], but this phenomenon of good health at low cost was recognised more than three decades ago [4, 5] and revisited more recently [6]. The two previous ‘good health at low cost’ studies focused in particular on the role of high quality equitable health care coverage (particularly primary health care) and disease prevention as well as successful advocacy for social and political support for health. However, neither analysis provided detail of the broader processes that may be implicated. In addition, the Institute for Health Metrics and Evaluation has published detailed quantitative analysis that shows new countries are emerging as punching above their weight, and while it identifies policies that have contributed to improvements, it again does not explore the wider context in detail [7].

So whilst these existing studies provide tantalising insights into the complex interactions between the characteristics of health care systems and the wider social, economic and political determinants of health that may underpin the unexpectedly positive (or negative) health outcomes achieved by some countries/regions, the picture is incomplete [8]. It is, therefore, vital to address these gaps in our knowledge if we are to improve the health and wellbeing of populations across the world. Doing so is timely, because at least nine of the seventeen Sustainable Development Goals for 2030 [9] require progress in aspects of health that extend beyond the boundaries of health systems, including the alleviation of poverty, hunger, and violence against women, as well as the provision of access to safe water and sanitation, labour rights, disaster preparedness, and action to combat climate change [10]. Further analysis of the countries that have and/or are punching above or below their economic weight more holistically is likely to offer important lessons.

## Establishment of the punching above their weight research network

It was in order to flesh out the contours of a wider conceptual and analytical framework that the international *Punching Above Their Weight Research Network* was established. It comprises researchers, policy actors and civil society representatives (all authors). The first meeting was held at the Rockefeller Bellagio Centre in August, 2017. The purpose of the Network is provided in Table 2.

**Table 2:** Punching Above Their Weight Research Network Purpose

1. Develop the capacity of members (by sharing knowledge and technical skills) to conduct research to understand why countries punch above or below their weight 2. Cement collaborative relations needed to undertake the research across country contexts 3. Develop research questions and specific hypothesis (based on theory and literature reviews) related to the complex interplay of political factors, governance regimes, civil society action, and specific policies (including the role of the health system) that contribute to the ability of a country/region to ‘punch above its weight’ 4. Develop methodological frameworks to allow investigation of these questions including empirical work on the most appropriate way to identify a diverse sample of countries that have or are punching above their weight and appropriate ‘matched’ comparators. 5. Disseminate findings of research to international agencies, national and regional governments and civil society.	

**Fig. 1:**
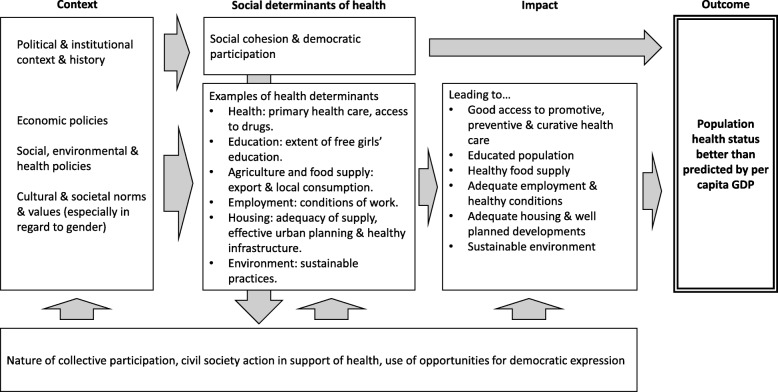
Preliminary framework to investigate why some countries punch above their weight in terms of health

Our discussions focused on the broad structural determinants of health in multiple countries/regions, seeking to understand the socioeconomic and political contexts known from research to drive population health and health equity. We also drew from the wider literature on the progress of nations in order to (i) identify issues absent from earlier studies or in need of further elaboration; and (ii) to develop preliminary research questions that would direct a broader investigation. The framework that developed from our discussions is shown in Fig. 1.

Our discussions highlighted the need to tease apart and closely examine the complexities involved in a country/region being able to punch above or below its weight in health terms, to determine what factors play a role, in what contexts, and to what effect. A number of particularly important and/or neglected complexities were identified.

## Future research agenda

Studies of the political and institutional context and history, a deeper understanding of policies on inequities, including gender, a more refined picture of disease burdens and improved measures of national performance were identified as being crucial to a future research agenda to understand the dynamics of what enables a country to do well in terms of life expectancy. Each are elaborated on below.

### Political and institutional context and history

A full understanding of the geographic, environmental sustainability, demographic, political, social and cultural context of a country is an essential background to understanding why it is ‘over’ or ‘under’ performing in health terms relative to its economic status. While the Commission on the Social Determinants of Health [11] improved understanding of some of these dynamics, further work using a complex systems analysis would be helpful [12]. This approach would enable unpacking of the relationships between different explanatory variables and enable whole-of-context understanding. Social determinants interact in complex systems so that “patterns of population health outcomes are an emergent property of the system. They arise from a web of causations that result from interactions among dynamic sets of interconnected systems” [13]. Such research needs to extend beyond epidemiology to include political science and sociological analysis. Such work must also consider diversity within countries. This is well exemplified by the Indian states, which have considerable political autonomy and very different health profiles and contexts [14]. More complex time-series work is required to track the progress of countries over time, allowing greater understanding of the contribution of age, period, and cohort effects and their causes. Some countries show steady upward progress (e.g. Sweden, Nepal) while others a more punctuated and gradual gain (e.g. Rwanda, Bangladesh). Others improve in a sprint and then may even decline (e.g. Zimbabwe). Work by Cardona and Bishai [15] suggests that economic growth is not associated with improved life expectancy when a time series analytical approach is used.

To understand the influence of context, the role of civil society action in raising awareness and creating democratic demand for improved health should be considered. Particular attention should also be devoted to the political conditions and dominant power relationships that have given rise to good population health. Szreter [16, 17] has conducted such research in relation to nineteenth century England to explain how health improved following the industrial revolution and provides insight into the political, economic and social insights required to understand population health. The discussions at the Bellagio meeting recognised that the political determinants of good health have rarely been unpacked and are still not adequately understood. The Lancet- University of Oslo Commission added some broad understanding of the global context and the implications of its work for countries remains to be determined [7, 18]. Methods to support research seeking to understand complex interactions will be multi-disciplinary drawing on social science and public health methodologies. They will be a mix of quantitative and qualitative methods. Quantitative data is required to describe the patterns of population health change including its distribution across different groups in the population and how it changes over time and to describe changing social and economic conditions (e.g. unemployment levels and literacy rates). Qualitative data can describe dynamics such as the distribution of power between groups and the extent to which civil society was able advocate for health promoting policies in different political contexts.

### Policies affecting socio-economic inequities

We need a deeper understanding of the role of inequities in resource distribution on overall population health. Within populations, there are often large variations in health among groups defined by income, wealth, ethnicity, race, education, and other characteristics. So far, most research has focused on measures of socio-economic position in high-income countries [19, 20] but further fine-grained analysis of the pathways by which these effects occur is required, especially in low and middle-income countries.

### Gender

Homogenous measures of life expectancy need to be broken down by sex, with further investigation required on the role of gendered power dynamics in influencing wellbeing. This will involve examining how gender roles, gendered opportunities and gender based discrimination influence health outcomes in countries, studying how these dynamics change over time to produce better or worse health for population groups. Within this analysis, the complexity of relationships needs to be accommodated to examine, for example, how female empowerment may lead to both positive and negative health impacts for women, such as by increasing women’s access to education but also exposing women to cultural and marketing pressures that may increase the likelihood that they will engage in unhealthy activities such as drinking and smoking. It is also important to look at the reasons why life expectancy among men is much lower than women in some countries.

### Disease burdens

Each country has a unique and changing disease burden [21], which reflects many factors, including its context and stage of development. Careful description and analysis of this burden (types of infectious versus non-communicable disease) and the responsiveness of particular types of disease to different determinants is required, including the magnitude of effects, and the time scale over which they occur. As an example, a significant proportion of the reduction in mortality in children under five in Brazil has been attributed to the increase in female literacy [22].

### New measures

Improved measures of progress that go beyond GDP are required. More sophisticated measures, such as the Genuine Progress Indicator (GPI) [23, 24] (which places more emphasis on things that people value, including leisure and volunteering and environmental impact) or the Index of Sustainable Economic Welfare [25] (which measures the depletion of resources) may facilitate understanding of health improvement. While such measures have been developed for high income countries [26, 27] they are also needed for low and middle income countries, and their association with health requires exploration. In addition, more carefully refined measures of health are required to consider the distribution of health *within* populations.

## Conclusion

The Bellagio meeting proposed that while previous research had provided a partial list of ingredients for good health, the complete recipe, listing all the ingredients and explaining how best they should be combined was not yet available. Our *Punching Above Their Weight Research Network* intends to build on the work described here and develop and conduct research within the framework presented in Fig. 1. We consider it essential to conduct this detailed political, social and economic analysis because the way research is framed also shapes the responses of policy makers and political actors to population health. Maintaining the current useful, but incomplete, knowledge base will restrict the political and policy actions of the future to a limited focus on health care reforms (important as these are) leaving the underlying drivers for effective population health action hidden.
